# Conflicts of interests, confidentiality and censorship in health risk assessment: the example of an herbicide and a GMO

**DOI:** 10.1186/s12302-014-0013-6

**Published:** 2014-06-24

**Authors:** Gilles-Eric Séralini, Robin Mesnage, Nicolas Defarge, Joël Spiroux de Vendômois

**Affiliations:** 1 Institute of Biology, EA2608, Network on Risks, Quality and Sustainable Environment MRSH-CNRS, University of Caen, Esplanade de la Paix, 14032 Caen Cedex, France; 2 CRIIGEN, 40 rue Monceau, 75008 Paris, France

**Keywords:** Conflicts of interests, Confidentiality, Retraction, GMO, Roundup, Glyphosate, NK603

## Abstract

We have studied the long-term toxicity of a Roundup-tolerant GM maize (NK603) and a whole Roundup pesticide formulation at environmentally relevant levels from 0.1 ppb. Our study was first published in *Food and Chemical Toxicology* (FCT) on 19 September, 2012. The first wave of criticisms arrived within a week, mostly from plant biologists without experience in toxicology. We answered all these criticisms. The debate then encompassed scientific arguments and a wave of *ad hominem* and potentially libellous comments appeared in different journals by authors having serious yet undisclosed conflicts of interests. At the same time, FCT acquired as its new assistant editor for biotechnology a former employee of Monsanto after he sent a letter to FCT to complain about our study. This is in particular why FCT asked for a *post-hoc* analysis of our raw data. On 19 November, 2013, the editor-in-chief requested the retraction of our study while recognizing that the data were not incorrect and that there was no misconduct and no fraud or intentional misinterpretation in our complete raw data - an unusual or even unprecedented action in scientific publishing. The editor argued that no conclusions could be drawn because we studied 10 rats per group over 2 years, because they were Sprague Dawley rats, and because the data were inconclusive on cancer. Yet this was known at the time of submission of our study. Our study was however never attended to be a carcinogenicity study. We never used the word ‘cancer’ in our paper. The present opinion is a summary of the debate resulting in this retraction, as it is a historic example of conflicts of interest in the scientific assessments of products commercialized worldwide. We also show that the decision to retract cannot be rationalized on any discernible scientific or ethical grounds. Censorship of research into health risks undermines the value and the credibility of science; thus, we republish our paper.

## Background

There is an ongoing debate on the potential health risks of the consumption of genetically modified (GM) plants containing high levels of pesticide residues [1]. Currently, no regulatory authority requests mandatory chronic animal feeding studies to be performed for edible GMOs and formulated pesticides. This fact is at the origin of most of the controversies. Only studies consisting of 90-day rat feeding trials have been conducted by manufacturers for GMOs. Statistical differences in the biochemistry of treated rats versus controls may represent the initial signs of long-term pathologies [2], possibly explained at least in part by pesticide residues in the GM feed. This is why we studied the long-term toxicity of a Roundup-tolerant GM maize (NK603) and a whole Roundup pesticide formulation at environmentally relevant levels from 0.1 ppb.

We first published these results in *Food and Chemical Toxicology* (FCT) on 19 September, 2012 [3] after a careful and thorough peer review. However, 1 year and 2 months later, in an unusual step, the editor-in-chief requested the retraction of our study, while conceding that the data were not incorrect and that there was no misconduct and no fraud or intentional misinterpretation. According to him, some data were inconclusive, but for reasons already known at the time of submission of the paper. The present paper is a summary of the debate resulting in this retraction, which in our view is a historic example of conflicts of interests in the scientific assessments of products commercialized worldwide.

## The long-term toxicity study of the NK603 maize and Roundup

An initial study on NK603 maize was submitted by Monsanto Company in support of commercial authorization of the maize. NK603 maize was fed to 4 groups of 20 Sprague Dawley rats (2 doses of 11% and 33% in the diet of both sexes) for 90 days [4]. The blood analyses were performed on 10 rats per group. The re-analysis of the raw data resulted in a debate on the biological relevance of admitted statistical differences versus controls as the first signs of hepatorenal toxicities [5]. To solve the problem, a 2-year-long study was carried out using two hundred Sprague Dawley rats to which the following treatments were administered: NK603 maize treated or not with Roundup at three different levels in their feed (11%, 22%, and 33% of the total diet) and Roundup alone, administered *via* drinking water at three different concentrations, from the admitted residual level in regular tap water (0.1 ppb), to the maximum level authorized in GMOs (400 ppm), up to half of the agricultural dose (0.5%). They were divided into ten groups, each containing ten males and ten females. No other long-term study has examined the effects of regular consumption of Roundup-tolerant GM maize and of a pesticide formulation, in any dilution, on blood parameters, sexual hormones, and multiple organs.

We found that these products provoked statistically discriminant disturbances in biochemical markers of livers and kidneys in females at the 15th month, when most of the rats were still alive. At the same time, testosterone and estradiol levels were also disturbed. At the end of the experiments, these disrupted biochemical markers corresponded to pathologies evidenced in a blinded manner: notably hepatorenal deficiencies, more severe in males, and female mammary tumors, which led to premature deaths. For instance, after around 700 days, there were up to 3.25 more mammary tumors (the highest rate was observed in females consuming 0.1 ppb of Roundup in water). This could be associated with a 2.4-time increase in pituitary dysfunctions noticed by the end of the experiment (2 years).

These findings were immediately dismissed by persons involved in the products’ authorizations, or in collaboration with biotech industries. A number of them wrote to FCT to nourish a controversy, including Richard Goodman, a former Monsanto employee in charge of the immunotoxicity files of GMOs, and Paul Christou, a patent holder of the methods used to create transgenic plants. This was rapidly followed by a coordination of national regulatory agencies organized by the European Food Safety Authority (EFSA), released on 4 October, 2012 [6]. The EFSA had previously assessed NK603, and glyphosate, the declared active principle of Roundup, as safe on the basis of regulatory data, which they never fully published. The EFSA has since published Monsanto’s safety data on NK603 maize [7], but not on glyphosate. The NK603 data are in a pdf format preventing an easy statistical re-analysis. However, there was no long-term toxicological assessment for NK603, or for Roundup. Moreover, we demonstrated in several studies [8-10] that Roundup is far more toxic than glyphosate because of non-inert adjuvants. On 10 October, 2012, the Monsanto Company also sent its criticisms to FCT [11] but did not release its safety data, claiming commercial confidentiality.

Overall, the first wave of criticisms arrived within a week, mostly from plant biologists. We answered all criticisms [12] in FCT on 9 November, 2012. The debate then encompassed scientific arguments. A second wave of *ad hominem* and potentially libelous comments appeared in different journals [13-16]. Regrettably, there were no invitations to respond to these exacerbated attacks, which we discovered only by our literature survey. Some of the authors of these articles had serious yet undisclosed conflicts of interest. The scientific remarks concentrated on the supposedly inadequate choice of the Sprague Dawley rat strain, which is, however, a classic model for toxicology [17]. The Sprague Dawley strain was also used by Monsanto in its 90-day test on the same maize [4]. In addition, Monsanto measured biochemically the same number of rats per group as in our experiment. Thus, with regard to blood and urine biochemistry, Monsanto gathered data from the same number of rats that we did.

## Unsubstantiated allegations of fraud or errors

Paul Christou, the lead author of Arjo et al. [13], demanded that our paper be retracted and insulted us personally. He claimed first in a letter addressed to the editor-in-chief that the publication of our study ‘*does not meet minimal acceptable standards of scientific rigor*’ and ‘*will damage an entire scientific discipline due to flawed conclusion*’ (personal communication). Then, he attacked us in an article published in the journal *Transgenic Research* on 20 December 2012 [13]. The quantity of insults and defamations in this paper, authorized and co-authored by the editor-in-chief in a supposedly serious journal, is excessive. They include: ‘*abject failure to treat the experimental animals in a humane manner*’, ‘*inability to formulate a valid hypothesis*’, ‘*media fanfare*’, ‘*fraudulent or knowingly inaccurate statements*’, ‘*unethical behavior*’, ‘*transparent attempt to discredit regulatory agencies*’, ‘*ammunition for extremists*’, ‘*flawed science*’, ‘*disingenuous or inept*’, and ‘*unjustified waste of animals*’ (while at the same time asking for more animals in the groups). Christou and co-authors suggest that by practising ‘*flawed science*’, we are working against ‘*progress towards a better quality of life*’ and in fact are ‘*actively working to make life worse*’. We were not invited to reply. This behaviour can be explained, though not justified, by the undisclosed conflicts of interests.

Christou is not only the editor-in-chief of Transgenic Research, the journal in which he published his article, but is also linked to Monsanto [18]. He is named as the inventor on several patents on GM crop technology, for most of which Monsanto owns the property rights. These include patents on the plant transformation process [19] used to make glyphosate-tolerant transgenic corn plants [20]. He worked as a researcher at Agracetus Inc. (later acquired by Monsanto) for 12 years. Then, from 1994 to 2001, Christou worked at the John Innes Centre in the UK [18], which is heavily invested in GM crop technology [21]. He thus has no mammalian toxicology background. However, in his published article, Christou only gave as his affiliation his publicly funded position at a research institute. Christou’s failure to declare his current interests - his inventor status on patents concerning the company that developed the products we tested - could be considered grounds for retraction of a paper in a scientific journal, according to ethical guidelines for scientific publishing [22].

The Arjo et al. article was co-authored by Wayne Parrott, an active member of the Biotechnology Committee at the International Life Sciences Institute (ILSI) [23]. ILSI is funded by multinational food, agribusiness, and biotechnology companies, including Monsanto and Syngenta [24]. ILSI has proved highly controversial in North America and Europe due to its influence on risk assessment methodologies for chemicals, pesticides, and GM foods [25-27]. Wayne Parrott also has an inventor status in patents on materials and methods for selecting transgenic organisms [28] and transformation vector systems [29].

In addition, Christou and his co-authors made numerous mistakes, false and unsubstantiated assertions, and misrepresentations of our data. The title of Arjo et al.’s paper includes defamation and a misrepresentation of our research, implying that it is ‘*pseudoscience*’ and alleging that it claimed Roundup Ready maize and Roundup herbicide caused ‘*cancer*’ in rats - a claim we never made. We did not even use the word ‘*cancer*’ in our paper although this argument was reiterated in the final letter of the editor-in-chief of FCT when explaining his decision to retract our paper [30]. Tumors do not always lead to cancer, even if they can be more deleterious in a shorter time because of their size or body position, by hurting internal functions.

Arjo et al.’s paper begins with a false assertion that is not evidenced in the paper or in the cited source: ‘*It started with a press conference in which journalists agreed not to engage in fact-checking*’. The authors made other false assertions about our study, for example, alleging that ‘*the water consumption was not measured*’. In fact, we measured both the water and food consumption, and the stability of the Roundup solution over time. This was indicated in the paper, in which we explained that all the data cannot be shown in one paper and that we concentrated on the most important data; these parameters were only part of a routine survey. They also falsified the reporting of the data, compiling the mortality data only at the end of the experiment and ignoring the originality and the major findings of the differential chronological effects between treated rats and controls, which we established by measuring tumor size twice a week over 2 years. Moreover, we respected legal requirements and ethical norms relating to animal experiments, and Arjo et al. present no evidence of the contrary, so their allegation of inhumane treatment of the rats is without substance.

Importantly, we had already answered many of the criticisms of our paper made by Arjo et al. in a paper that was published before that of Arjo et al. [12]. Their publication was received on 20 December 2012, when our paper was published on 9 November 2012. Our published answers were simply ignored.

Christou was not alone in failing to declare conflicts of interest in his criticism of our paper. Since we underlined that 75% of the comments addressed to FCT within a week after our study was published came from plant biologists, it was discovered that several had developed patents on GMOs. Some authors were employees of Monsanto Company, which owns NK603 GM maize and sells Roundup herbicide [4,11]. Other more recent papers, published by plant biologists and/or affiliates of the industry-funded group ILSI [15,16], repeated the arguments. The author of a separate article criticizing our study expressed concern that our results could damage public opinion about GM crops [14] - a sentiment that gives precedence to economic interests over public health. An article in Forbes magazine even alleged, without presenting any evidence, that we had committed fraud [31]. Surprisingly, even Monsanto authors [11] declared that they had ‘no conflicts of interest’ in their first draft published online on FCT website. Investigative reports [32,33] evidenced that many authors of these opinions had failed to disclose their conflicts of interest, including Henry Miller, Mark Tester, Chris Leaver, Bruce Chassy, Martina Newell-McGloughlin, Andrew Cockburn, L. Val Giddings, Sivramiah Shantharam, Lucia de Souza, Erio Barale-Thomas, and Marc Fellous. The undisclosed conflicts of interest included links with biotechnology companies that develop GMOs and with industry-backed lobbying organizations.

All of this has huge implications for public health. We observed an intense lobbying in parliaments, as well as proofs of conflicts of interests for persons involved in the regulatory decisions for the commercialization of these products [26]. A series of high-profile conflict-of-interest revelations (not restricted to GMOs and pesticides) led to the resignations of leading administrators involved in decisions affecting the assessment of these products, including the European Commissioner John Dalli [34] and the former chair of the European Food Safety Authority’s (EFSA) management board Diana Banati [35]. In February of 2013, a strange occurrence following the publication of our paper raised questions about the connections of industry to scientific publishing, described below.

## Conflicts of interests in the editorial board

In February 2013, FCT acquired a new assistant editor for biotechnology, Richard E. Goodman. The editor-in-chief has admitted that Goodman was introduced into the editorial board after he sent a letter to FCT to complain about our study. In his letter, Goodman appears worried about economic consequences but not so much about potential public health consequences (personal communication). He wrote: ‘*The implications and the impacts of this uncontrolled study is having HUGE impacts, in international trade, in consumer confidence in all aspects of food safety, and certainly in US state referendums on labelling*’. Further in his letter, Goodman asked for ‘*an evaluation by an independent set of toxicologists*’. This is particularly why the Publishing Assistant for FCT asked for our raw data on 15 March 2013.

In fact, we can question the independence of this re-evaluation. After his appointment at FCT, Goodman was a member of the subcommittee that requested our raw data, until we complained to Elsevier publishing group. Goodman is far from being independent. He previously worked for Monsanto for 7 years [36]. He also has a long-standing affiliation with ILSI [37]. Goodman will now deal with all biotechnology papers submitted to FCT. Another scientific paper on GMO risks was withdrawn from FCT, without explanation shortly after it had been accepted and published by the journal [38]. The paper was immediately published by another journal [39] according to the authors’ initiative.

We received a letter from the editor-in-chief of FCT, A. Wallace Hayes, asking us to retract our paper on 19 November 2013, more than 1 year after its publication [40]. In his retraction notice, the editor-in-chief certifies that ‘*no evidence of fraud or intentional misrepresentation of the data*’ was found in the investigation, that the results are ‘*not incorrect*’, ‘*there was no misconduct*’, and that the sole reason for retraction is the ‘*inconclusiveness*’ of the paper. He argued that no conclusions could be drawn because we studied 10 rats per group over 2 years, because they were Sprague Dawley rats, and because we could not conclude on cancer. In fact, the Sprague Dawley is a standard choice for 2-year studies performed by industry and independent scientists alike [17,41]. We also measured 10 animals per sex per group according to OECD 452 guideline on chronic toxicity studies [42] because our study is a chronic toxicity study that was never intended to be a carcinogenicity study. We wish to point out that Dr Hayes’ decision is in violation of the retraction guidelines of the Committee on Publication Ethics (COPE), of which FCT is a member. ‘*Inconclusiveness*’ is not a valid reason for a journal to retract a paper. Lack of conclusiveness (which can be discussed) and error are not synonymous. COPE criteria for retraction included scientific misconduct/honest error, prior publication, plagiarism, or unethical research. None of these criteria applied to our study. On the contrary, numerous published scientific papers contain inconclusive findings. It is for further studies to build on the reported findings and arrive at a more conclusive position. In contrast with our study measuring toxicity, the Monsanto study reporting safety with the same number and the same strain of rats, but limited to 90 days, [4] is not subject to the same controversy. The data in the Monsanto study show statistically significant differences in multiple-organ functions between the GM and non-GM feeding groups, which the authors dismissed as not ‘biologically meaningful’, using a set of questionable criteria [43]. The significant effects observed do not have to be linear to the dose to be taken into consideration; otherwise, endocrine effects will be dismissed. In addition, biochemical disturbances do not have to correlate simultaneously with organ lesions, in contrast to the claims of Doull et al. [44] in defence of Monsanto. These outdated concepts coming from the toxicology of poisons, and are not valid for endocrine disruption [43,45]. If 10 rats/sex/group are too few to demonstrate a toxic effect, then this number of rats is certainly too small to demonstrate safety. Overall, in the current system of assessment, any toxic effect is first suspected to be a false positive, arising by chance, rather than questioning whether no evidence of effect is a false negative result. The Monsanto data as presented are thus inconclusive and should also be retracted.

Following the retraction of our paper, many letters were sent to the editor-in-chief of FCT. On 10 December 2013, he published a defence of the retraction, which raised many doubts as to his understanding of our data [30]. He claimed that we concluded on cancer, although ours was a long-term toxicity study with a detailed statistical analysis of blood and urine parameters. He also defended the study done by Monsanto [4] claiming that they used 20 rats/sex/group while we only used 10 rats/sex/group. In fact, despite the fact that the Monsanto study used twice our sample size, the Monsanto authors only analyzed blood and urine from half of the animals (10), the same number of sampled animals as in our study.

According to an editorial in Environmental Health Perspectives [46], ‘*the decision to retract a published scientific work by an editor, against the desires of the authors, because it is ‘inconclusive’ based on a post hoc analysis represents a dangerous erosion of the underpinnings of the peer-review process, and Elsevier should carefully reconsider this decision*’.

## Confidentiality and censorship erode the value of science

Recent reviews of the GM food safety literature have found that research concluding that GM products were safe tended to come from industry and that research conducted by those with either financial or professional conflicts of interest was associated with outcomes favorable to the GM sector [47]. In fact, it appears in our case that consequences of conflicts of interests in science go beyond divergence in scientific interpretations and also rely on unscientific practices: confidentiality and censorship.

Transparency of, and access to, all the raw data obtained by companies and accepted by regulatory agencies (overall blood analyses of rats) as proof of safety for products, is an unavoidable first step to move forward in this debate. It is the only way in which the scientific community can enter the scientific discussion. This is why we republish our paper in an open access way, together with its raw data allowing debate about our results. This is not possible for the data used as a proof of safety for commercial authorizations. The Monsanto toxicological data on NK603 maize recently made public by EFSA is not in a statistically usable format and an agreement with Monsanto is requested before use. Moreover, the data examined for Roundup authorizations are clearly inadequate [48]. For instance, ANSES (French Agency for Food, Environmental and Occupational Health & Safety), confirmed to us in writing (January 2013) that there were no 2-year studies of Roundup in its whole formulation on animals, adding that there are a few studies of acute toxicity (a few days up to 3 weeks) without any blood tests. Instead, glyphosate, which is much less toxic than Roundup [10,49], is tested alone by Monsanto, in its reports to regulatory authorities [50]. We strongly emphasize that data with implications for public health are not related to manufacturing patents and should not be kept confidential. Removal of confidentiality claims on biosafety data is necessary to adhere to standard scientific procedures of quality assurance, to increase transparency, to minimize impacts of conflicts of interests, and ultimately to improve public confidence in GMOs [51]. Moreover, in the regulatory assessment of GMOs, chemicals, and medicines, confidential tests are conducted by the applicant companies themselves, often in their own laboratories or in those of subcontractors.

The second step must be the building of new experiments for new or the most important products, by laboratories independent of the companies. They will be recruited by public tender, with compulsory transparency of the results. This public research will be funded by companies, at a level corresponding to their previous budget for regulatory testing, but managed independently of the companies. The protocols and results will be submitted to open and contradictory assessments. Thus, there will be no additional financial cost or time delay to the current system. Such reforms will not only radically transform the understanding and knowledge of toxicology and science in general, but will radically reduce public health costs and promote trust in companies and science. This will move the world towards a sustainable development of products with low, if any, impacts on health and environment.

The reason given to retract our paper - ‘*inconclusiveness*’ - is unprecedented and violates the norms of scientific publishing. The decision to retract cannot be rationalized on any discernible scientific grounds. Censorship on research into the risks of a technology so critically entwined with global food safety undermines the value and the credibility of science.
